# Antibody titers and rapid antigen testing in elderly patients with SARS-CoV-2 pneumonia vs. staff of ICU and “Covid-19” wards

**DOI:** 10.3205/dgkh000382

**Published:** 2021-03-15

**Authors:** Jörg Epstude, Marcin Skiba, Igor Alexander Harsch

**Affiliations:** 1 Department of Hospital Hygiene, Thuringia Clinic “Georgius Agricola”, Saalfeld/Saale, Germany; 2 Department of Internal Medicine II, Thuringia Clinic “Georgius Agricola”, Saalfeld/Saale, Germany

**Keywords:** antibodies, COVID-19, SARS-CoV-2, serology, rapid antigen testing

## Abstract

**Aim:** The majority of patients hospitalized with COVID-19 are older individuals. Age and the comorbidities typically associated with it usually go hand in hand with a less favorable course of the disease. We were interested in the antibody response in this particular patient group as well as in the results of rapid antigen testing.

**Methods:** In 30 elderly patients (>75 years), antibody titers (IgA and IgG) against COVID-19 were measured, and rapid antigen testing was determined about 3 weeks after the onset of symptoms of SARS-CoV-2 infection. The results were compared with those of a “high-risk” group consisting of “Covid-19” ward regular staff, as well as with “low-risk” staff consisting of members of the intensive care unit (ICU). The antibody titer against SARS-CoV-2 was determined by ELISA (EUROIMMUN™, PerkinElmer, Inc. Company); for rapid antigen testing, we used the SARS-CoV-2 Rapid Antigen test (Roche^®^).

**Results:** Our investigations demonstrate a robust antibody response in the majority of elderly, comorbid patients about three weeks after the onset of infection. At this timepoint, most of the results of rapid antigen testing were negative. Furthermore, in the group of employees of our clinic (“Covid-19” ward vs. the ICU staff), the prevalence of antibodies was very low and antigen testing was negative in the whole ICU group.

**Conclusion:** Although frequently comorbid, elderly patients are capable of significantly increasing antibodies against COVID-19 about 3 weeks after the onset of infection. Since the viral load can be assumed to have been low at that point, rapid antigen testing was negative in most cases. In the test group of employees of our clinic (“Covid-19” ward vs. the ICU staff), the data demonstrate that – given adequate protective measures – the risk of infection is not higher in a “Covid-19” ward compared to other wards.

## Zusammenfassung

**Ziel:** Die Mehrheit der mit COVID-19 ins Krankenhaus eingelieferten Patienten ist im höheren Lebensalter. Das Alter und die typischerweise damit verbundenen Komorbiditäten sind mit einem ungünstigeren Krankheitsverlauf verbunden. Wir waren an der Antikörperantwort in dieser Patientengruppe sowie an den Ergebnissen von Antigen-Schnelltests interessiert.

**Methode:** Bei 30 älteren Patienten (>75 Jahre) wurden Antikörpertiter (IgA und IgG) gegen COVID-19 bestimmt und der Antigen-Schnelltest ungefähr 3 Wochen nach Auftreten der Symptome der SARS-CoV-2 Infektion bestimmt. Die Ergebnisse wurden mit denen einer „Hochrisikopopulation“ verglichen, die aus regulärem Personal einer „Corona“-Station besteht, sowie mit einem „Niedrigrisiko“-Personal, das aus Mitgliedern der Intensivstation besteht. Die Antikörperbestimmung gegen SARS-CoV-2 wurde mittels ELISA (EUROIMMUN™, PerkinElmer, Inc. Company) durchgeführt. Für die Antigen-Schnelltests verwendeten wir den SARS-CoV-2-Rapid-Antigen-Test (Roche^®^).

**Ergebnisse:** Unsere Untersuchungen zeigen eine „robuste“ Antikörperantwort bei der Mehrzahl der älteren, komorbiden Patienten etwa drei Wochen nach Beginn der Infektion. Die meisten Ergebnisse von Antigen-Schnelltests waren zu diesem Zeitpunkt negativ. Darüber hinaus war in der Gruppe der Mitarbeiter der Klinik („Corona“-Station im Vergleich zum Personal auf der Intensivstation) die Prävalenz von Antikörpern sehr gering und die Antigentests in der gesamten Gruppe auf der Intensivstation negativ.

**Schlussfolgerung:** Obwohl häufig komorbid, können ältere Patienten etwa 3 Wochen nach Beginn der Infektion einen signifikanten Anstieg der Antikörper gegen SARS-CoV-2 verzeichnen. Da die Viruslast zu diesem Zeitpunkt als gering angenommen werden kann, war der Antigen-Schnelltest erwartungsgemäß in den meisten Fällen negativ. In der Gruppe der Mitarbeiter der Klinik („Corona“-Station im Vergleich zum Personal auf der Intensivstation) zeigen die Daten, dass bei angemessenen Schutzmaßnahmen das Infektionsrisiko in einer Station für Corona-Erkrankte im Vergleich zu anderen Stationen nicht höher ist.

## Introduction

The majority of patients hospitalized with a COVID-19 infection are older individuals. Age and the typical comorbidities that come with it are associated with a less favorable course of the disease [1].

Recently, Nikolich-Zugich et al. [2] summed up their review about SARS-CoV-2 and COVID-19 in older adults with the statements that the immune response in older adults is slower, less coordinated, and less effective. Furthermore, they reported evidence that the immune response to SARS-1 does not effectively switch from innate to adaptive (little or no antibody production) immunity and speculated that this may also be the case in a SARS-CoV-2 infection.

With the beginning of a “second wave” of the COVID-19 pandemic in Europe, not only antibody testing is widely available in clinical routine, but also rapid antigen tests have become part of our diagnostic tools. Several authors, however, regard this testing as more of an adjunct to RT-PCR testing because of the higher potential for false-negative results [3], [4].

We took the opportunity to study the presence and levels of antibodies in elderly patients and also to evaluate rapid antigen testing in this group. Very recently, the German health minister signed a “Corona Ordinance”. Covid-19 tests are now to be concentrated more on risk groups and the healthcare system and less on returning travelers. Among other things, it was planned that the Corona Ordinance called for nursing homes and hospitals to be enabled to make extensive use of “rapid antigen tests” so that visitors, staff and patients could be tested regularly and quickly [5]. We used these tests for evaluation purposes in our patient group (see above).

For comparative purposes, we also determined the presence and levels of antibodies in a “high-risk” population consisting of the same “Covid-19” ward regular staff reported about in our study on the “first wave” [6], as well as in “low-risk” staff consisting of members of our intensive care unit (ICU); in the latter, rapid antigen testing was performed. During the “first wave”, 23 patients with a COVID-19 infection were hospitalized in the COVID-19 regular ward. Three of them also had to be placed in the intensive care unit, but only for a few days, which is why we regard this staff as a “low-risk” group.

## Methods

### Study participants and procedure

After obtaining informed consent, and with approval by the Ethics Committee of the State Medical Association of Thuringia, we examined the IgA and IgG antibody titers of the intensive care staff (n=50) and the COVID-19 regular staff (n=18) (both consisting of doctors, nurses and cleaning personnel) in October 2020, as well as those of 30 elderly patients with a COVID-19 infection (>75 years, range 75–93 years) in our clinic from October to November 2020, including additional rapid antigen testing. The testing was done three weeks after the onset of clinical symptoms of a COVID-19 infection (see discussion for rationale). In the first patients (n=5), the infection was related to a stay in a Czech Spa and medicinal baths; in the others, the infection was most likely acquired within the family.

The diagnosis of COVID-19 infection was established via oropharyngeal smears using multiplex real-time PCR with three specific gene probes (N, E, and RdRp). The abbreviations refer to structural proteins of the coronavirus. These are the nucleocapsid protein (N), the small envelope protein (E) and RNA-dependent RNA polymerase (RdRp). The detection limit is 100 RNA copies/reaction.

### Rapid Antigen Test 

The SARS-CoV-2 Rapid Antigen test (Roche^®^) principle is that of a chromatographic immunoassay for the detection of viral envelope antigens of SARS-CoV-2 in the nasopharynx. On a nitrocellulose membrane, the test has two precoated lines, a control line and a test line, and delivers results after 15–30 minutes. The distributor reports a sensitivity of 96.52% and a specificity of 99.68%.

### Antibody measurement 

An enzyme-linked immunosorbent assay (ELISA) is used for determining antibodies against SARS-CoV-2 (EUROIMMUN™, a PerkinElmer, Inc. company). According to the distributor, the specificity of the test for IgG is 98.5% and 92.5% for IgA. Antibody titers below 0.8 are negative, and we consider titers 2 and above to be reliable and significant.

## Results

Anthropometric parameters as well as the results of the testing are presented in Table 1 (Tab. 1). 

The burden of comorbidity in the 30 elderly patients consisted of

- heart failure (8; 26.6%),
- hypertension (22; 73.3%),
- coronary heart disease (12; 40%),
- stroke (4; 13.3%),
- diabetes mellitus (14; 46.6%),
- kidney disease (8; 26.6%),
- inflammatory bowel disease (2; 6.6%), and
- cancer in remission (1; 3.3%).

**Figure 1:**
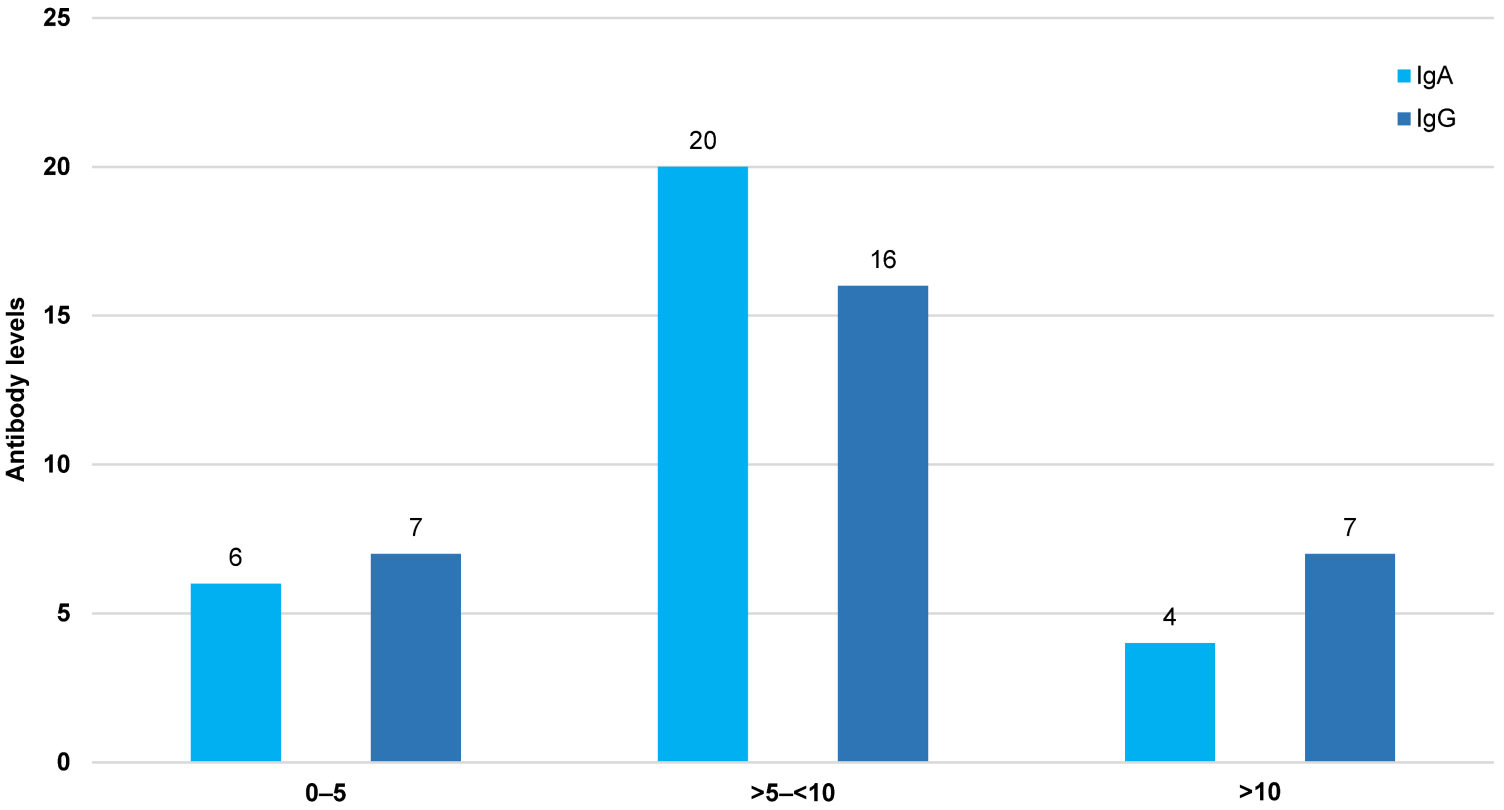
Antibody levels in patients >75 years about three weeks after onset of symptoms (patient numbers are given on top of the columns)

Twelve patients (40%) had a BMI >30 kg/m^2^. The antibody levels are given in Figure 1 (Fig. 1).

## Discussion

In the elderly patients, antibody testing was done about three weeks after the onset of clinical symptoms of COVID-19 infection. This time point was chosen since the initial subgroup of elderly patients investigated here was often admitted to the hospital about 2 weeks after onset of symptoms (when we initially also measured these antibodies) due to problems such as lack of access to everyday items (e.g., food) and care or delayed convalescence. The repeated antibody testing in this patient group revealed a robust antibody response about 3 weeks after the onset of clinical symptoms (fever, respiratory symptoms) or most likely after contact with the source of infection.

The measurements revealed relatively high titers of COVID-19 antibodies, which does not support the hypothesis that elderly patients might have a problem in this regard. However, the robust titer levels do not necessarily contradict Nikolich-Zugich et al. [2] that the immune response in older adults is slower and less effective. The number of tested patients is not high and the burden of comorbidities was relatively low in this group. Patients with active cancer were not included. Diabetes mellitus, which had a high prevalence, has been proposed as a major risk factor for an unfavorable course of COVID-19 infection [7], [8]. But recently, an observational study by Lampasona et al. [9] demonstrated that a humoral response against SARS-CoV-2 in patients with diabetes was present and highly comparable (as for timing and antibody titers) to that of non-diabetic patients, with only marginal differences, and was not influenced by glucose levels. The average age in the group with diabetes mellitus investigated was 69 (58–76) years.

It is also worth mentioning that all our patients survived the SARS-CoV-2 pneumonia, which is *per se* an indicator of good immune response. A decrease of the antibody titers can be expected in the further course. It is not yet known whether the peak levels of the antibodies are of prognostic value concerning protection from further infection.

As for the results of rapid antigen testing, it lies in the nature of an immunoassay to be “better” when a higher antigen load is present. As expected, in patients admitted to the clinic with an onset of symptoms 2–3 weeks prior to admission, and thus, a relatively low viral load, most of these test results were negative. The “real” value of rapid antigen testing can be expected in patients with a very recent onset of symptoms and a high viral load. According to our observations, we were usually able to generate more positive results in the rapid test up to a week after the onset of symptoms (data not shown). Other authors also reported a poor performance of rapid antigen detection test as frontline testing for a COVID-19 diagnosis, also due to sensitivity problems [4]. It would be of interest to examine whether the results of the rapid antigen tests correlate with the Ct (cycle-threshold) value of quantitative RT-PCR as a potential parameter for virus load and infectiousness as suggested by La Scola et al. [10]. However, this parameter is not yet provided by most labs on a routine basis.

In the group of clinic employees examined for comparative purposes, there were almost no antibodies against COVID-19. This applied to both the staff in the “Covid-19 ward” and the staff in the intensive care unit; the latter were only confronted with a very small number of these patients and only for short periods of time. The fact that antibodies were only present in a few employees is an expression of the relatively low incidence of the disease in Thuringia at the time of our study, but also demonstrates that the risk of infection for employees in “Covid-19 wards” is not higher than that of employees in other wards. However, the low incidence of such antibodies also shows that the staff in these wards are immunologically “unprotected” in the face of the second wave, which emphasizes the continued need for strict hygiene measures.

Recently, it was also confirmed in a larger number of study participants (n=660) that clinic employees in “Covid-19 wards” do not have a higher risk of infection than other employees in clinics [11].

## Conclusions

Our study demonstrates a robust antibody response in elderly, comorbid patients about three weeks after onset of infection. In patients with an onset of symptoms 2–3 weeks prior to admission, and presumably a relatively low viral load, most results of rapid antigen testing were negative. Furthermore, in the group of employees of our clinic (“Covid-19 ward” vs. ICU staff), the prevalence of antibodies was very low, demonstrating that – given adequate protective measures – the risk of infection is not higher in a “Covid-19” ward than in other wards.

## Notes

### Competing interests

The authors declare that they have no competing interests.

### Acknowledgements

We are grateful to the persons who provided blood samples to support scientific research and to Andrea Ortloff for the graphics and the organisatorial support.

We are grateful to M. Reinhöfer (DIANOVIS) for providing antibody measurements in the control groups free of charge.

## Figures and Tables

**Table 1:**
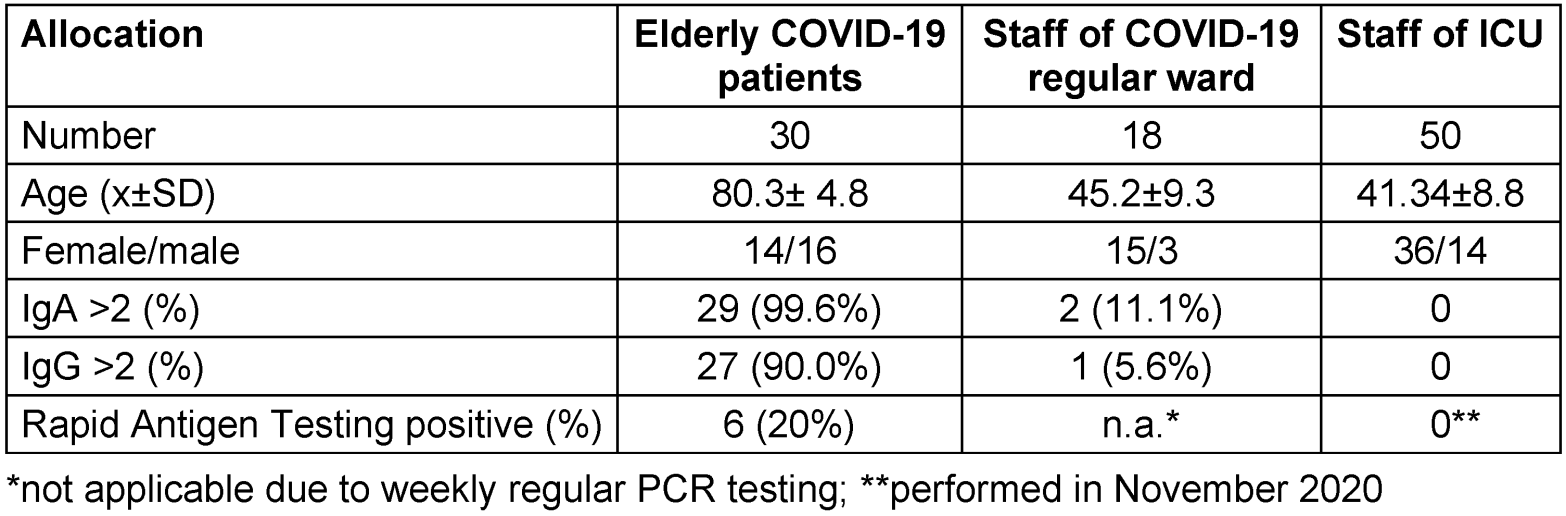
Anthropometric parameters and presence of antibody titers in the personnel and the elderly patients
